# High-throughput microbial population genomics using the Cortex variation assembler

**DOI:** 10.1093/bioinformatics/bts673

**Published:** 2012-11-19

**Authors:** Zamin Iqbal, Isaac Turner, Gil McVean

**Affiliations:** ^1^Wellcome Trust Centre for Human Genetics, Roosevelt Drive, Oxford, OX3 7BN, UK and ^2^Department of Statistics, South Parks Road, Oxford, OX1 3TG, UK

## Abstract

**Summary:** We have developed a software package, Cortex, designed for the analysis of genetic variation by *de novo* assembly of multiple samples. This allows direct comparison of samples without using a reference genome as intermediate and incorporates discovery and genotyping of single-nucleotide polymorphisms, indels and larger events in a single framework. We introduce pipelines which simplify the analysis of microbial samples and increase discovery power; these also enable the construction of a graph of known sequence and variation in a species, against which new samples can be compared rapidly. We demonstrate the ease-of-use and power by reproducing the results of studies using both long and short reads.

**Availability:** http://cortexassembler.sourceforge.net (GPLv3 license).

**Contact:** zam@well.ox.ac.uk, mcvean@well.ox.ac.uk

**Supplementary information**: Supplementary data are available at Bioinformatics online.

## 1 INTRODUCTION

Genome sequencing of pathogens is of growing value to public health as a means of distinguishing genetic variants correlated with drug resistance (Mwangi *et al.*, 2007) or virulence (den Bakker *et al.*, 2011), for tracking of transmission chains, and for within-host evolution studies (Young *et al.*, 2012). At the heart of all these applications is the requirement to be able to compare the genomes of different samples. Most current tools (Li *et al.*, 2009; McKenna *et al.*, 2010) are dependent on alignment to a reference genome, and cannot detect differences between genomes directly. It is known that alignment introduces a bias towards the reference (Degner *et al.*, 2009) and has limited power for indel discovery [Fig. 3a in Iqbal *et al.* (2012)]. Furthermore, using a reference is inherently problematic as microbial strains can be highly diverged.

We showed in Iqbal *et al.* (2012) that the Cortex assembler has power to detect single-nucleotide polymorphisms (SNPs), indels and structural variants by simultaneously *de novo* assembling multiple samples (each corresponding to a ‘colour’ in a de Bruijn graph) and detecting variants as graph motifs. A limitation of a de Bruijn graph approach is the use of a single kmer size. There is in general no optimal kmer for variant discovery in a given dataset—variants near repeat sequence will only be discoverable at higher kmers, but lower coverage variants will need a lower kmer [(Iqbal *et al.*, 2012) Supplementary Information Section 1.3 onwards]. We have therefore introduced pipelines to do discovery on many samples at many kmer values, and then genotype all samples at all discovered sites. Discovery can be done jointly in all samples (with or without a reference), or per sample against a reference. We demonstrate by reproducing the results of two published studies.

## 2 JOINT OR PER-SAMPLE DISCOVERY

**Fig. 1.**
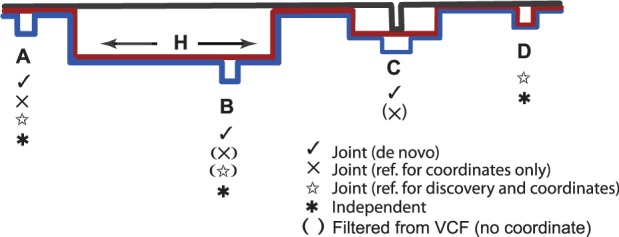
Cartoon shows two samples (red and blue) and a diverged reference genome (black) in a de Bruijn graph; symbols indicate which workflows call sites. Brackets indicate flanking regions cannot be placed on reference, so the call is filtered from VCF. At SNP B, the independent workflow would call the red and blue versions of H and would implicitly find B. C is only called if the reference is excluded from discovery, as it confounds the red allele. D is called by workflows that include the reference in discovery

The new Cortex pipelines take sequence data as input (in FASTQ, FASTA or BAM format) and build and clean per-sample graphs for multiple kmer values. The *joint workflow* then uses a multi-colour graph to directly compare all samples using the Bubble Caller algorithm (Iqbal *et al.*, 2012); discovery and genotyping of biallelic sites are done at the same time. A reference can be incorporated (a) not at all (in which case the VCF uses dummy coordinates), (b) for coordinates only or (c) in discovery as well as for coordinates (Fig. 1). Option (a) allows calling of sites where both alleles are non-reference (sites B and C in Fig. 1); (a) and (b) avoid any confounding effects of reference sequence divergence or reference errors (site C in Fig. 1) and (c) allows calling of monomorphic non-reference variants (site D in Fig. 1).

In contrast, the *independent workflow* calls variants independently for each sample, for each kmer value in a two-colour sample-plus-reference graph [using either the Bubble Caller or Path Divergence Caller (Iqbal *et al.*, 2012)], and genotypes all samples at all sites in a single traversal of a multi-colour graph. In combining biallelic per-sample calls, this can detect overlapping and multiallelic sites. See examples of these workflows in Fig 1.

For eukaryote genomes, variant assembly sensitivity is limited by coverage and repeat content. This effect is reduced for microbial genomes (less repetitive, deeper sequencing), and assembly can have comparable power to mapping approaches (Supplementary Information Sections 2 and 4 and Case Study 2). False-discovery rate is independent of workflow, and driven by sequencing errors and repeats (Supplementary Information, Section 4).

## 3 SCALING TO THOUSANDS OF SAMPLES

Cortex memory-use scales linearly with number of kmers and samples (Supplementary Information, Section 1). Error cleaning is done per sample, so sequencing errors have no impact on subsequent stages. Therefore, memory use peaks when all samples are loaded into the graph for discovery/genotyping. Processing 1000 *Staphylococcus aureus* samples would require of the order of 106 Gb RAM (Supplementary Information, Section 5). Time complexity of each step is shown in Table 1; note the non-parallelizable steps scale linearly with number of samples.

**Table 1.** Time complexity of workflow stages

Step	Complexity	1000 samples/ 100 CPUs	Parallelizable
Build	O(D)	45 min	I,J
Error clean	O(G)+O(D)	3.3 min	I,J
Load all samples	ϵc	40 min (1 CPU)	no
Discovery	O(G)	2 min	I
Genotyping	O(S) + O(cμ)	8 min (1 CPU)	no

D = depth of coverage, c = number of colours/samples, G = genome size, S = number of sites, μ = mean allele length, ϵ = time to load one sample graph (2.5 s for *S.aureus*), I = independent, J = joint. Column 3 gives estimates for analysis of 1000 *S.aureus* samples with 100 CPUs and the Bubble Caller, based on Case Study 2 (8 min based on fact that genotyping of 72 samples in Case Study 2 took 35 s).

## 4 A REPOSITORY OF VARIATION

Assembled graphs and callsets are a repository of known sequence and variation. If a new sample is obtained, pre-existing binaries (both workflows) and callsets (independent) are reused; the sample is compared with previous sequence (joint) or with the reference (independent) and all samples are regenotyped at all sites. Thus, Cortex allows new samples to be compared with the entire pan-genome (see Supplementary Information Section 13 for further details).

## 5 CASE STUDIES

### 5.1 Rapid high-quality calls without manual curation

We show Cortex can generate results of comparable quality to those from labour-intensive whole-genome assembly and alignment. In Mwangi *et al.* (2007), the authors Sanger-sequenced two strains of *S.aureus* (depth 9×), did two whole-genome assemblies, mapped reads to contigs, a three-way alignment between the assemblies and a reference, called variants, and removed alignment errors, giving 32 validated SNP differences on the chromosome. Post-publication, 8 further SNPs (one validated) were found when the assemblies were finished (M. Mwangi, personal communication). Using the joint workflow (*k* = 31,61 and 160 MB RAM), it took 188 seconds to call 36 differences between the strains, including 29 (2) of the published (post-publication) Mwangi callset, 30 of the 33 validated calls and 33 confirmed by finished references (Supplementary Information Section 11).

### 5.2 Comparable sensitivity to mapping

We here show Cortex attaining the same sensitivity as mapping-cased calling for microbial genomes by reproducing the results of (Young *el al.*, 2012), who analysed 72 isolates (100× each, 100 bp Illumina reads) of *S.aureus*. They used mapping (Stampy/samtools) to two references (MSSA476 and their own assembly), plus variant assembly (early release of Cortex), plus manual curation, to find 32 (2) SNPs/indels placeable on MSSA476 (their reference). We reproduced their 32 calls (independent workflow, kmer = 61, 10 GB RAM), in 5.5 h (one CPU). The other two sites were also called (independent+their reference or joint workflow) (Supplementary Information Section 12).

## 6 CONCLUSION

We have shown that the Cortex population assembler provides a method for rapid, high-quality variant discovery in microbial genomes and allows comparison of new samples with all previously known strains. We believe it will prove valuable in microbial population genomics.
